# Supplementary material for: Associations of alcohol consumption and physical activity with lean type 2 diabetes mellitus among Korean adults: A prospective cohort study
Source: PLoS One. 2020 Sep 3;15(9):e0238641. doi: 10.1371/journal.pone.0238641 (PMC7470281; doi:10.1371/journal.pone.0238641)
Supplement: Attachment — Submitted filename: Responses to Reviewers_2v.docx [file pone.0238641.s002.docx]

Re: PONE-D-20-03085
Associations of alcohol consumption and physical activity with lean type 2 diabetes mellitus among Korean adults: A prospective cohort study

Here are our responses to the comments for the above-referenced manuscript.

Response to the Reviewer #1’s comments:

Reviewer #1:

1. I asked the authors to explain in the introduction, why it is important to investigate the incidence of T2DM among lean individuals. The only explanation they give is that there is not much previous evidence on this topic. However, I think just the fact that there is not much evidence does not justify the topic. So, why is it important to investigate the incidence of T2DM among lean individuals?

Response: Thank you for further comments. We have now added contents related to the importance of research for lean T2DM (lines 58-60 and 70-72, revised).

2. Reviewer 2 brought up an excellent point about non-response. In the revised version, the authors state that “Because the health surveys were administrated by trained researchers, the number of non-responders was trivial”. As long as a survey is voluntary, there is a significant risk that non-random non-response occurs. The authors should provide exact information concerning non-response.

Response: We have now added the exact proportion of non-responders (1.2%) (line 299, revised).

Reviewer #2: I am happy with the revised version of the paper. I like the research question, the structure of the paper, the quality of writing, and the way the authors describe their empirical proceeding and results. Most importantly, the authors have addressed all the issues stated in my referee report for the first version appropriately.

Response: Thank you very much for your valuable comments.
